# Targeting cardiovascular and metabolic risk modification in end stage renal disease (ESRD): a randomized controlled clinical trial on niacin’s effects on lipoprotein(a) and biochemical markers in hemodialysis patients

**DOI:** 10.3389/fmed.2025.1625417

**Published:** 2025-10-09

**Authors:** Amira Reda Muhammad Galal, Maha Abdel Rhman Salah, Ammena Y. Binsaleh, Nawal Alsubaie, Amani S. Alrossies, Ahmed Hassan Elthakaby, Ghadir Mohamed Elsawy, Azza A. Ali, Zeinab Al Kasaby Zalat

**Affiliations:** ^1^Clinical pharmacy Department, Faculty of Pharmacy (Girls), Al-Azhar University, Cairo, Egypt; ^2^Department of Pharmacy Practice, College of Pharmacy, Princess Nourah Bint Abdulrahman University, Riyadh, Saudi Arabia; ^3^National Institute of Urology and Nephrology, Cairo, Egypt; ^4^Pharmacology and Toxicology Department, Faculty of Pharmacy (Girls), Al-Azhar University, Cairo, Egypt

**Keywords:** niacin, hemodialysis, end-stage renal disease patients, lipoprotein (a) concentration, hyperphosphatemia, hyperuricemia

## Abstract

**Background:**

End-stage kidney disease (ESRD) patients on dialysis face pronounced cardiovascular and metabolic risks due to disruptions in lipoprotein(a), phosphorus, potassium, uric acid, and lipid balance. Current therapeutic options offer limited capacity to address these multifaceted abnormalities. Niacin is unique in this regard, as it not only lowers lipoprotein(a) but also influences phosphorus and uric acid metabolism. This study evaluates the efficacy of niacin therapy in improving these biochemical markers, thereby addressing an important therapeutic gap in this vulnerable population.

**Methods:**

In a randomized, controlled trial, 50 hemodialysis patients were divided into two groups of twenty-five each. The control group continued standard care, while the niacin group received 500 mg/day niacin alongside standard therapy. Patients were followed for 3 months.

**Results:**

Systolic and diastolic blood pressure were stabilized by niacin administration, in contrast to the control group, where both parameters rose significantly. Phosphorus decreased significantly in the niacin group (5.59 to 4.85 mg/dL, *P* = 0.0077), while increasing in controls. PTH levels decreased by 60% with niacin but rose by 41.6% in controls (*P* = 0.001). Potassium levels fell by 27% in the niacin group, whereas they rose by 23.9% in controls. Sodium remained stable with niacin but declined in controls. Uric acid levels rose sharply in controls but remained stable with niacin. Niacin significantly improved lipid profiles, notably reducing LDL (31.9% vs. 7.9%) and total cholesterol (13.3% vs. 3.7%). Although triglycerides and VLDL rose in both groups, these variations were not of statistical significance. Importantly, Lp(a) levels decreased by 11.4% in the niacin group.

**Conclusion:**

Niacin (500 mg/day) offers significant cardiovascular and metabolic benefits for hemodialysis patients, supporting its role as an adjunctive therapy in managing ESRD-associated risks.

**Clinical trial registration:**

https://clinicaltrials.gov/, NCT06406140

## 1 Introduction

Clinically, chronic kidney disease (CKD) is a recognized condition characterized by sustained impairment in renal structure and/or function, with an irreversible and progressive trajectory. It is widely prevalent among the adult population (1).

Clinically, end-stage renal disease is classified as CKD stage G5, which necessitates the start of renal replacement therapy since eGFR, or glomerular filtration rate, is below 15 milliliters per minute (1.73 cubic meters) (2).

Global prevalence of ESRD has significantly intensified, positioning it as a critical public health issue, primarily attributable to the rising life expectancy across the globe. Currently, over 2.5 million individuals receive renal replacement therapy, with this number expected to double by 2030. Patients with ESRD face a markedly elevated risk of cardio-cerebrovascular disease (CVD), which serves as an independent predictor for the initiation of dialysis. Dyslipidemia, along with frequent fluctuations in lipid and lipoprotein profiles, has been recognized as one of the primary causes of cardiovascular disease’s onset and progression in the impacted population (3).

Patients diagnosed with ESRD undergoing hemodialysis treatment exhibit markedly elevated levels of lipoprotein(a) [Lp(a)] in the blood compared to healthy individuals. Furthermore, those receiving maintenance hemodialysis present Lp(a) concentrations that are approximately five to ten times greater than levels observed among individuals with chronic renal disease in its early stages, highlighting a substantial increase associated with advanced renal failure and its treatment modalities. The increased cardiovascular risk seen in this population is thought to be exacerbated by this increase in Lp(a) (4).

One important and distinct risk factor for both cardiovascular and cerebrovascular disorders is lipoprotein (a). However, it is still unclear exactly which pathways lead to higher Lp(a) serum levels in CKD, the kidney has been considered to be involved in the catabolic clearance of this lipoprotein (5).

The lipid core of lipoprotein (a), which is made up of triacylglycerols and cholesteryl esters, is surrounded by a surface layer of phospholipids, unesterified cholesterol, and apolipoprotein B-100 (apoB-100). This structure is similar to that of low-density lipoprotein (LDL). Apolipoprotein(a) [apo(a)], a glycoprotein that is covalently bonded to apoB-100 by a single disulfide bond, is its distinctive component. This unique structural feature imparts distinct biological properties to Lp(a), influencing its role in atherogenesis and cardiovascular risk (6).

In addition to having a high cholesterol content, elevated serum [Lp(a)] has the potential to cause atherosclerosis because it resembles LDL particles and shares structural similarities with plasminogen and plasmin, which have prothrombotic and antifibrinolytic effects (7).

As the glomerular filtration rate (GFR) gradually declines, the kidney’s capacity to maintain electrolyte homeostasis becomes increasingly impaired. Consequently, electrolyte imbalances—such as hyperphosphatemia, often accompanied by elevated serum parathyroid hormone levels—commonly arise in patients undergoing hemodialysis (8).

The parathyroid glands release the polypeptide hormone known as parathyroid hormone (PTH), which is essential in modulating the homeostatic balance of calcium and phosphate via influencing the gastrointestinal system, renal tubules, and bone. In CKD, the renal capacity to synthesize active vitamin D is reduced as a result of the enzyme 1-alpha-hydroxylase’s lower activity, which is ordinarily upregulated by PTH to convert 25-hydroxyvitamin D into its biologically active form, 1,25-dihydroxyvitamin D. The resulting deficiency in active vitamin D diminishes intestinal calcium absorption, triggers compensatory PTH secretion, promotes parathyroid gland hyperplasia, and leads to the development of secondary hyperparathyroidism. In advanced stages of CKD, PTH becomes insufficient to facilitate adequate renal phosphate excretion, culminating in a biochemical profile characterized by hypocalcemia, hyperphosphatemia, and decreased levels of active vitamin D (9).

Hyperphosphatemia represents a common metabolic complication noticed in patients with ESRD, particularly those undergoing maintenance hemodialysis. This condition is strongly correlated with heightened morbidity and mortality, including an elevated risk of cardiovascular complications, and has also been associated with prolonged hospitalization in the hemodialysis population (10).

Although a variety of therapeutic strategies have been developed to manage hyperphosphatemia in individuals receiving dialysis for ESRD, current therapy options remain suboptimal. Calcium-based phosphate binders may lead to adverse effects, including hypercalcemia. In contrast, non-calcium-based binders such as sevelamer and lanthanum, while effective, are often limited by their high cost. Although aluminum-containing phosphate binders are efficacious, their use has markedly declined due to concerns regarding aluminum toxicity (11). Therefore, there is a critical need for novel therapeutic agents or adjunctive treatments to existing phosphate binders that enhance efficacy, demonstrate favorable tolerability, and offer cost-effectiveness (12).

Patients undergoing maintenance hemodialysis (HD) are more likely to experience hyperkalaemia, which is a condition where serum potassium levels above 5.0 mmol/L, despite adherence to a thrice-weekly dialysis regimen. Hyperkalemia represents a potentially fatal electrolyte disturbance, as it predisposes patients to cardiac arrhythmias and sudden cardiac arrest (13).

Human purine metabolism results in uric acid, which is mostly removed by the kidneys, which make up 60–70% of total excretion. The intestines then remove the remaining 30–40%. Hyperuricemia, a disorder marked by increased uric acid levels, is one of the many factors accelerating the development of ESRD, affecting between 40% and 80% of patients with ESRD, underscoring its significant role in disease advancement (8).

There are numerous cardiovascular diseases and hypertension associated with hyperuricemia. A substantial association has been observed between uric acid concentrations on the blood and the risk of mortality; and, specifically, each 1 mg/dL rise correlates with an 8% increase in the overall mortality risk and cardiovascular disease (14).

An inflammatory response, oxidative stress, and endothelial dysfunction associated with ESKD may be exacerbated by hyperuricemia when it is coexisting with ESKD. Cardiovascular disease, which continues to be the primary cause of death for individuals with ESKD, is largely caused by these mechanisms. Elevated uric acid levels have been demonstrated to be associated, and potentially predict, cardiovascular mortality in individuals undergoing hemodialysis. Therefore, effective reduction of uric acid is an important target for treatment in the treatment of ESKD, with the potential to substantially improve patient survival outcomes (15).

Vitamin B3, or nicotinic acid, is a naturally occurring, vitamin being soluble in water with numerous biological functions. Recent research has shown that active phosphate transport inhibitors can be effective as monotherapy or adjunct therapy for hyperphosphatemia. The niacin drug class represents a promising therapeutic option to reduce serum phosphates among hemodialysis patients, due to its unique ability to inhibit intestinal phosphate transport mechanisms (16).

Recent studies indicate that nicotinic acid and its derivative, nicotinamide (NAM), can reduce intestinal phosphorus absorption in animal models via a mechanism distinct from that of conventional phosphate binders. NAM prevents the renal proximal tubule’s sodium phosphate co-transporters and the intestinal NaPi2b co-transporters *in vitro* to decrease phosphate uptake. In a similar way to its inhibitory action on phosphate transporters, niacin primarily metabolizes to NAM, resulting in suppression of intestinal phosphate absorption (10).

Elevated fibroblast growth factor-23 (FGF23) levels represent an early adaptive response aimed at preserving phosphate excretion as glomerular filtration rate declines. Growing evidence associates increased circulating FGF23 with adverse outcomes, including progression of kidney disease and heightened cardiovascular risk. In addition to reducing serum phosphorus, extended-release niacin may offer further benefit by lowering circulating FGF23 concentrations (17).

It is emerging evidence that niacin has multiple benefits when it comes to CKD such as modulating dyslipidemia, reducing serum phosphate, improving endothelial function, and preventing inflammation and oxidative stress. The combination of these mechanisms makes CKD patients much less likely to suffer fatal outcomes from cardiovascular disease and all other causes (18).

Niacin has a major impact on lipid metabolism. Total cholesterol, low-density lipoprotein cholesterol (LDL-C), and triglyceride levels are considerably reduced while high-density lipoprotein cholesterol (HDL-C) is increased, as part of the wide range of pharmacological effects that niacin has on lipid metabolism. Niacin also significantly lowers plasma concentrations of Lp(a), a recognized significant risk factor for coronary heart disease, in addition to these lipid-modifying effects. Because of its many benefits, niacin is a useful medication for treating dyslipidaemia and lowering the risk of cardiovascular disease (18).

It has been demonstrated that niacin inhibits the transcriptional activity of the promoter of the LPA gene, which is responsible for encoding apolipoprotein(a), thereby contributing to the modulation of lipoprotein(a) levels (19). Additionally, niacin may influence the synthesis of apolipoprotein B100, a key component of lipoprotein(a) (20).

### 1.1 Aim of study

In the context of renal impairment, preliminary studies have explored the impact of niacin in managing hyperphosphatemia and dyslipidemia. Previous studies have predominantly focused on assessing how niacin affects lipid profiles and blood phosphorus levels in patients with chronic kidney disease in order to determine its influence on renal function.

However, despite growing interest in the pleiotropic effects of niacin, limited attention has been given to its impact in the dialysis patients. This study aims to evaluate the therapeutic potential of niacin as a multifaceted adjunct therapy in patients undergoing maintenance hemodialysis, with a particular focus on reducing cardiovascular risk and improving metabolic balance. By assessing its effects on lipoprotein (a), blood pressure, uric acid, lipid profile, phosphorous level, potassium level, sodium level, and parathyroid hormone level (PTH), the study seeks to give an expanded understanding of niacin’s role beyond lipid-lowering, targeting key biochemical markers linked to cardiovascular morbidity in dialysis patients. This represents a novel approach compared to earlier studies that primarily emphasized its impact on renal function. By broadening the scope of investigation, this study sought to offer a more thorough elucidation of the therapeutic potential of niacin in patients receiving hemodialysis.

## 2 Materials and methods

### 2.1 Study design and ethical approval

It was a randomized, prospective, parallel, and controlled clinical trial conducted at the National Institute of Urology & Nephrology (NIUN), Cairo, Egypt, in dialysis units. The trial was authorized by the Research Committee of the General Organization for Teaching Hospitals and Institutes (GOTHI) No. (IUN00040) and the Ethical Committee in the Faculty of Pharmacy (Girls), Al-Azhar University No. (452), and it is being conducted according to the 1964 Declaration of Helsinki.

### 2.2 Participant selection

**FIGURE 1:**
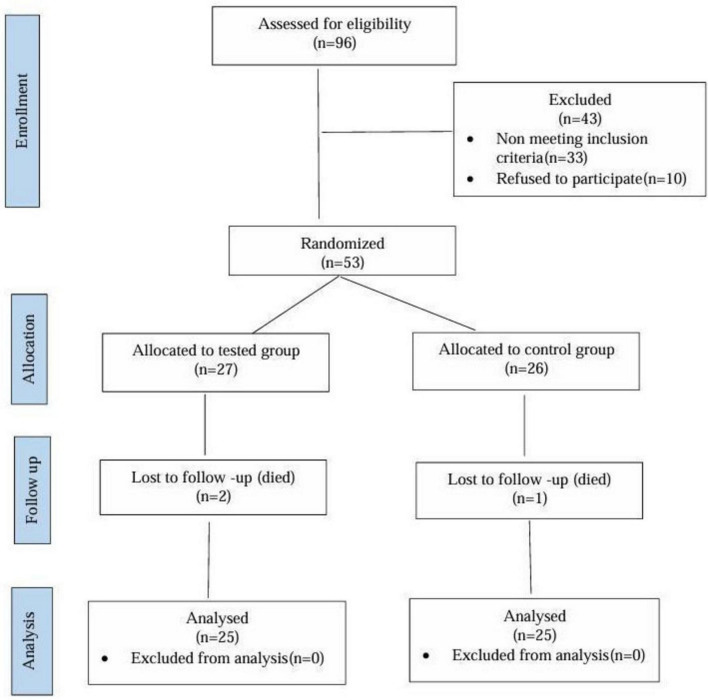
Flow diagram representation of the study design. n, number of patients.

From May 2024 to October 2024, a total of 96 patients in the National Institute of Urology & Nephrology (NIUN), Cairo, Egypt, in dialysis units, according to the study protocol’s specified inclusion and exclusion criteria, participants’ eligibility was assessed. As shown in the flow diagram representation of the study design (Figure 1), inclusion criteria were patients diagnosed with ESRD, participants had to be on maintenance hemodialysis for at least 3 months, aged 18 or older, both sexes, without any known contraindications to niacin therapy, and who had agreed to participate. Exclusion criteria were pregnant and breast-feeding women; extended-release niacin should not be used in patients with a medical condition or taking any medications that would contraindicate its use, such as active peptic ulcer disease. An adverse reaction to the study medication or an infection or acute gouty attack within 2 weeks before enrollment. The study excluded patients with chronic liver disease, immune-suppressive therapy, non-compliant patients, and those who failed to adhere to their medications.

### 2.3 Sample size calculation and randomization

The sample size was determined systematically to guarantee sufficient statistical power for addressing the study objectives. This calculation was performed by the Department of Community, Environmental, and Occupational Medicine, Faculty of Medicine, Ain Shams University, using Power Analysis and Sample Size Software (PASS 15, Version 15.0.10), with statistical power set at 80% and a significance level (α) of 0.05. Based on this estimation, the study required the inclusion of fifty patients CKD undergoing hemodialysis. Participants were allocated into two groups: a control group, which received only standard therapy for 3 months, and a niacin group, which was administered 500 mg of niacin daily in addition to standard therapy over the same duration.

### 2.4 Evaluation of clinical findings

All patients underwent assessments at baseline and after 3 months of treatment initiation. These evaluations included demographic and clinical history data collection, monitoring of adverse effects, assessment of treatment adherence, and a series of laboratory investigations (biochemical parameters). The measured variables comprised blood pressure, lipoprotein(a), uric acid, lipid profile, serum phosphorus, potassium, sodium, and parathyroid hormone (PTH) levels.

### 2.5 Study outcomes

Considering the recognized correlation between elevated Lp(a) levels and heightened cardiovascular risk in individuals with ESRD, the study’s major goal was to assess the change in Lp(a) levels after niacin treatment in patients undergoing maintenance hemodialysis. The secondary outcomes of the study were change in phosphorus levels after niacin supplementation, change in uric acid concentrations in response to niacin treatment due to their implications for cardiovascular and metabolic health in ESRD, change in potassium levels, to assess the risk of hyperkalemia—a common and life-threatening complication in hemodialysis patients and change in blood pressure measurements, including both systolic and diastolic values, to evaluate the impact of niacin on cardiovascular regulation. This comprehensive approach aims to facilitate a more comprehensive understanding of the multi-faceted benefits of niacin in ESRD management.

### 2.6 Statistical analysis

XLSTAT 2019 and SPSS version 27 (SPSS Inc., Chicago, IL, USA) were used for data analysis. Quantitative data were shown as mean ± standard error (SE), while qualitative variables were expressed as frequencies (N) and percentages (%). Data normality was assessed using the Shapiro-Wilk test. When data were normally distributed, the paired-sample *t*-test was used for intragroup comparisons of pre- and post-intervention values; for variables that were not normally distributed, the Wilcoxon signed-rank test was employed. The Mann-Whitney U test for non-parametric data and the independent-samples *t*-test for normally distributed data were used to evaluate differences between groups. Statistical significance was established at a significance level of 0.05, and *p*-values below this threshold were deemed significant.

## 3 Results

### 3.1 Baseline demographic data of study subjects

A total of 53 patients were enrolled and randomized into either the control or niacin groups. Three participants did not complete the study due to early mortality (two in the control group and one in the niacin group), leaving fifty patients who successfully completed the trial. Baseline demographic characteristics are presented in Table 1. The mean age was 46.12 ± 1.56 years in the control group and 50.96 ± 2.29 years in the niacin group, with a *P*-value of 0.089, indicating no significant age difference between the groups. Similarly, comparisons of gender distribution and body weight yielded *P*-values of 0.5443 and 0.796, respectively, confirming that both groups were well matched at baseline.

**TABLE 1:** Baseline demographic data.

Variable	Study groups		
	Control group N = 25	Intervention (Niacin) group N = 25	*P*-value
Age (years) Mean ± SE	46.12 ± 1.56	50.96 ± 2.29	0.089
Gender: male N (%) Female N (%)	7 (28%) 18 (72%)	9 (36%) 16 (64%)	0.5443
Weight (Kg) Mean ± SE	79.85 ± 2.830	78.53 ± 4.190	0.796

### 3.2 Patient’s baseline characteristics

Patient’s baseline characteristics (comorbid conditions and potential causes of hemodialysis) are shown in Table 2. All *p*-values were more than 0.05, which indicates the homogeneity of patient’s distributions in the study groups.

**TABLE 2:** Patient’s baseline characteristics (comorbid conditions, and potential causes of Hemodialysis).

Comorbid conditions	Control group N (%)	Intervention group N (%)	*P*-value
Hypertension	10 (40%)	10 (40%)	1.000
Diabetes mellitus	4 (16%)	6 (24%)	0.4795
Heart failure	5 (20%)	3 (12%)	0.4404
Hyperlipidemia	2 (8%)	3 (12%)	0.6374
Anemia	22 (88%)	18 (72%)	0.1573
**Potential causes of (HD)**	**Control group** **N (%)**	**Intervention group** **N (%)**	***P*-value**
Hypertension	10 (40%)	10 (40%)	1.000
Diabetes mellitus	4 (16%)	6 (24%)	0.4795
Glomerulonephritis	3 (12%)	2 (8%)	0.6374

### 3.3 Baseline comparison of clinical data and laboratory test results between control and intervention groups

Before the beginning of the study, we compared both groups as regards their clinical data and laboratory tests, as shown in Table 3. Both groups were matched, and no significant differences were detected between the groups, as all *p*-values exceeded the 0.05 threshold.

**TABLE 3:** Baseline comparison of clinical data and laboratory test results between control and intervention groups.

Variable	Normal range	Control versus intervention group at baseline			
		Control group N = 25 Mean ± SE	Intervention group N = 25 Mean ± SE	Test statistic	*P*-value
Systolic blood pressure (mmHg)	less than 120	135.32 ± 2.759	133.08 ± 4.458	−0.176	0.861
Diastolic blood pressure (mmHg)	Less than 80	81.16 ± 1.988	81.60 ± 2.340	0.178	0.859
Potassium (mEq/L)	3.5–5.1	7.04 ± 0.150	6.70 ± 0.200	−1.215	0.224
Phosphorus (mEq/dl)	2.5–5	5.81 ± 0.245	5.59 ± 0.198	0.712	0.480
Sodium (mEq/L)	136–145	135.80 ± 0.327	137.08 ± 0.568	−1.953	0.058
PTH (pg/ml)	10–65	488.40 ± 14.821	464.46 ± 24.282	−1.886	0.059
Uric acid (mg/dl)	2.4–6	6.07 ± 0.139	6.41 ± 0.257	−1.167	0.250
Triglycerides (mg/dl)	>150	131.34 ± 7.760	165.22 ± 15.096	−1.950	0.051
VLDL (mg/dl)	25–50	26.40 ± 1.537	29.91 ± 2.062	−1.931	0.053
Cholesterols (mg/dl)	Up to 200	176.02 ± 5.683	168.56 ± 5.576	0.938	0.353
HDL (mg/dl)	40–60	38.32 ± 0.899	33.70 ± 2.139	1.993	0.055
Non-HDL (mg/dl)	>130	136.36 ± 5.655	134.82 ± 5.649	0.193	0.848
LDL (mg/dl)	>100	111.18 ± 4.508	101.73 ± 4.810	1.433	0.158
Lipoprotein a (mg/dl)	Less than 30	145.42 ± 7.473	130.08 ± 8.239	1.379	0.174

### 3.4 Comparison of clinical data and laboratory test results between control and intervention groups at baseline and after 3 months of treatment

We examined the two groups 3 months after the study began in order to ascertain how niacin affected the following parameters:

#### 3.4.1 Effect on blood pressure

**FIGURE 2:**
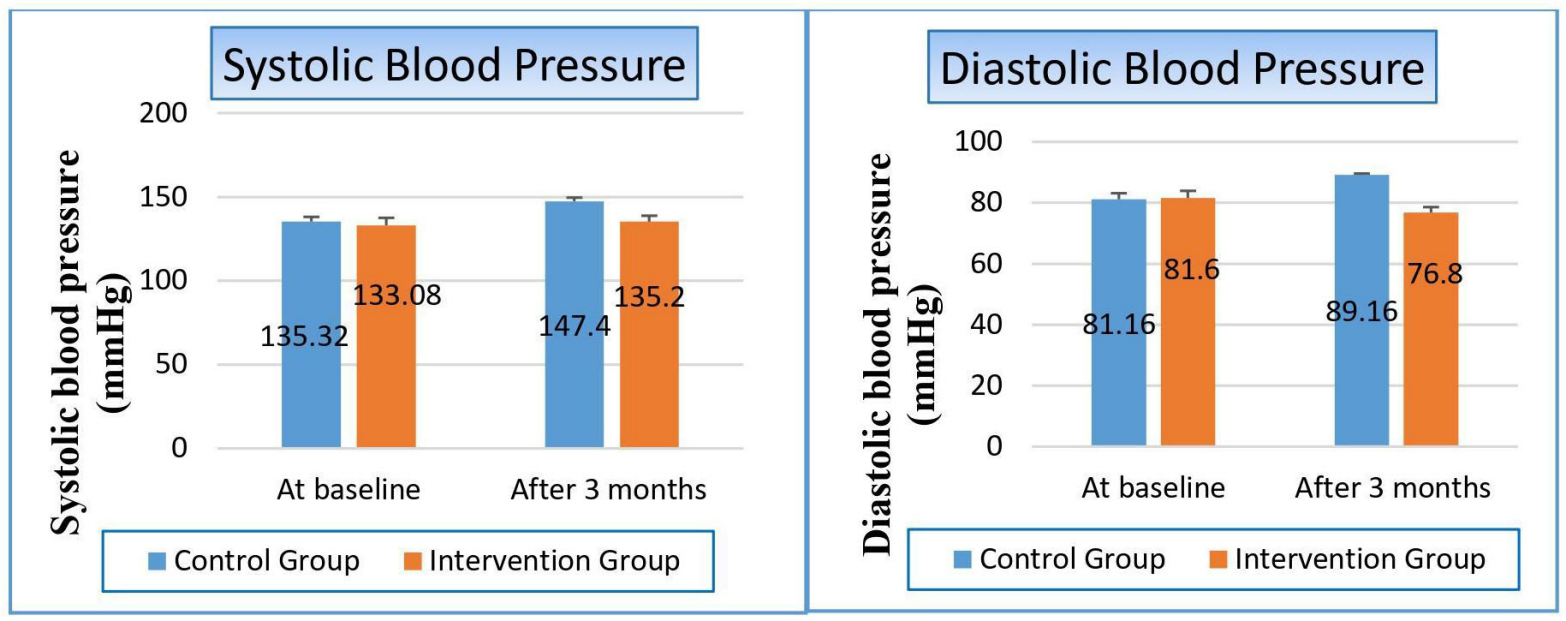
Changes in blood pressure in control versus intervention groups at baseline and after 3 months.

After 3 months, the control group demonstrated a significant rise in blood pressure, with systolic values increasing by 12.08 mmHg (9%) and diastolic values by 8 mmHg (9.6%), showing *P*-values of 0.000 and 0.002, respectively. In contrast, the niacin group exhibited no significant changes in either systolic or diastolic blood pressure following the intervention period. Nevertheless, when compared to the control group, the niacin group showed a marked improvement in both systolic and diastolic blood pressure, with *P*-values of 0.046 and 0.001, respectively (Figure 2 and Table 4). These findings highlight the beneficial impact of niacin on blood pressure regulation.

**TABLE 4:** The effect on blood pressure.

Variable	Study groups		
	Control group	Intervention group	*P*-value
**Systolic blood pressure (mmHg)**			
At baseline Mean ± SE	135.32 ± 2.759	133.08 ± 4.458	0.861
After 3 months Mean ± SE	147.40 ± 2.195	135.20 ± 3.576	**0.046**
T-statistics	−3.447	−0.316	
*P*-value	**0.000**	0.752	
**Diastolic blood pressure (mmHg)**			
At baseline Mean ± SE	81.16 ± 1.988	81.60 ± 2.340	0.859
After 3 months Mean ± SE	89.16 ± 0.450	76.80 ± 1.800	**0.001**
T-statistics	−3.098	−1.680	
*P*-value	**0.002**	0.093	

Bold values indicate statistically significant results (*p* < 0.05).

#### 3.4.2 Effect on serum electrolytes:

**FIGURE 3:**
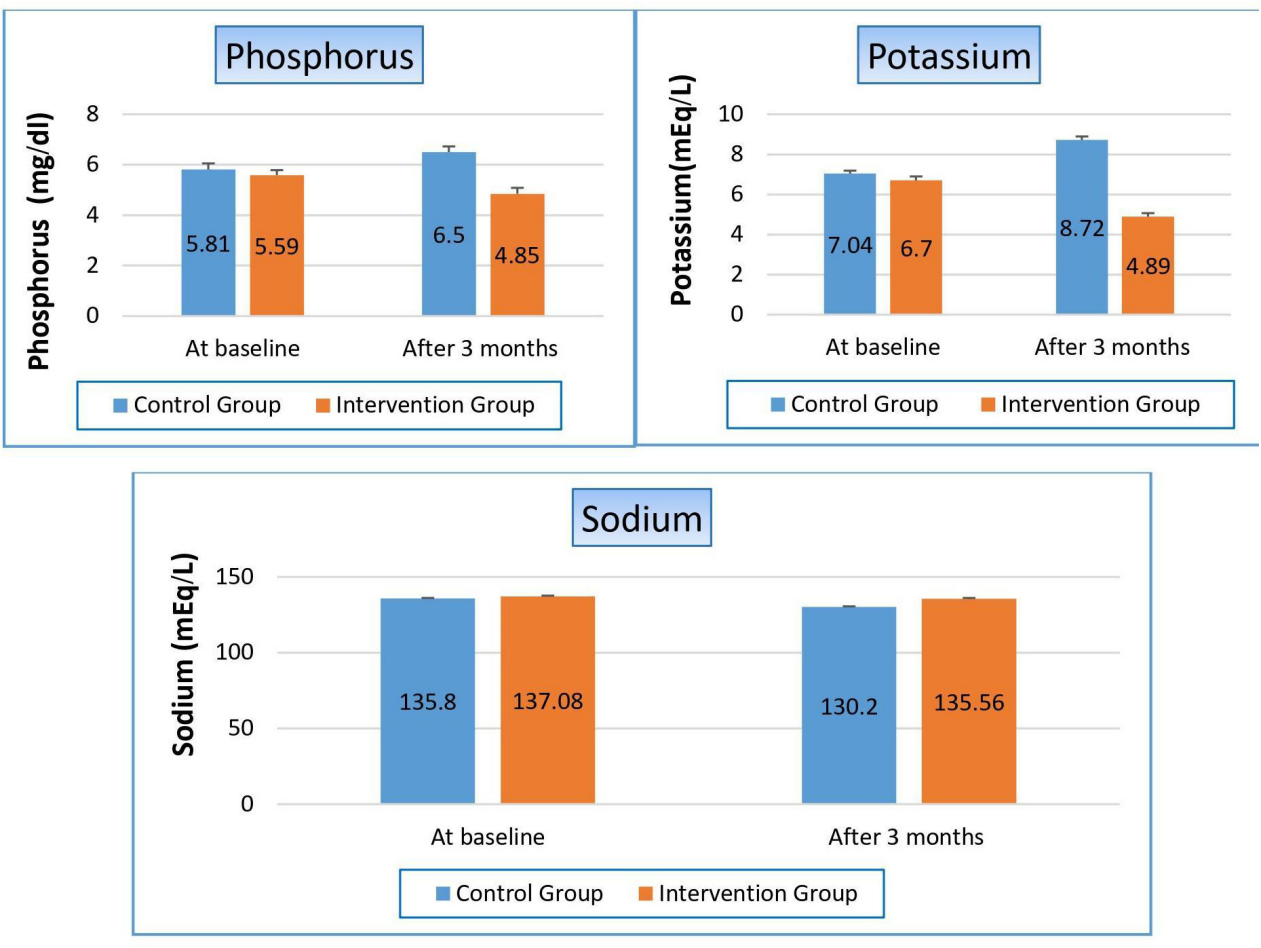
Changes in serum electrolytes in control versus intervention groups at baseline and after 3 months.

Patients within the control group suffered from a significant hyperphosphatemia by 11.9% (*p*-value 0.008). Conversely, however, there was a significant improvement in phosphorus level within the niacin group, where it reduced from 5.59 to 4.85 to become within normal range (*p*-value 0.0077). These results reflected positively on the *p*-value between both groups (*p*-value 0.000). The same observation occurred in potassium level, where controlled patients gained a significant hyperkalemia, which increased by 23.9% from baseline, while there was a substantial decrease in potassium level in the niacin group by 27%, so there was a highly significant reduction between both groups in favor of the niacin group (*p*-value 0.001). On the contrary, hyponatremia increased in the control group by 4.13%, while the decrease in sodium level was still within the normal range in the niacin group, which significantly appeared in the comparison between both groups (*p*-value 0.000). There were no notable variations between the two groups or within each group, as shown in Figure 3 and Table 5.

**TABLE 5:** Effect on serum electrolytes.

Variable	Study groups		
	Control group	Intervention group	*P*-value
**Phosphorus (mg/dl)**			
At baseline Mean ± SE	5.81 ± 0.245	5.59 ± 0.198	0.480
After 3 months Mean ± SE	6.50 ± 0.228	4.85 ± 0.235	**0.000**
T-statistics	−2.651	−3.44	
*P*-value	**0.008**	**0.0077**	
**Potassium (mEq/L)**			
At baseline Mean ± SE	7.04 ± 0.150	6.70 ± 0.200	0.224
After 3 months Mean ± SE	8.72 ± 0.176	4.89 ± 0.178	**0.001**
T-statistics	−4.345	8.250	
*P*-value	**0.000**	**0.000**	
**Sodium (mEq/L)**			
At baseline Mean ± SE	135.80 ± 0.327	137.08 ± 0.568	0.058
After 3 months Mean ± SE	130.20 ± 0.396	135.56 ± 0.563	**0.000**
T-statistics	−4.304	2.135	
*P*-value	**0.000**	**0.043**	

Bold values indicate statistically significant results (*p* < 0.05).

#### 3.4.3 Effect on uric acid

**FIGURE 4:**
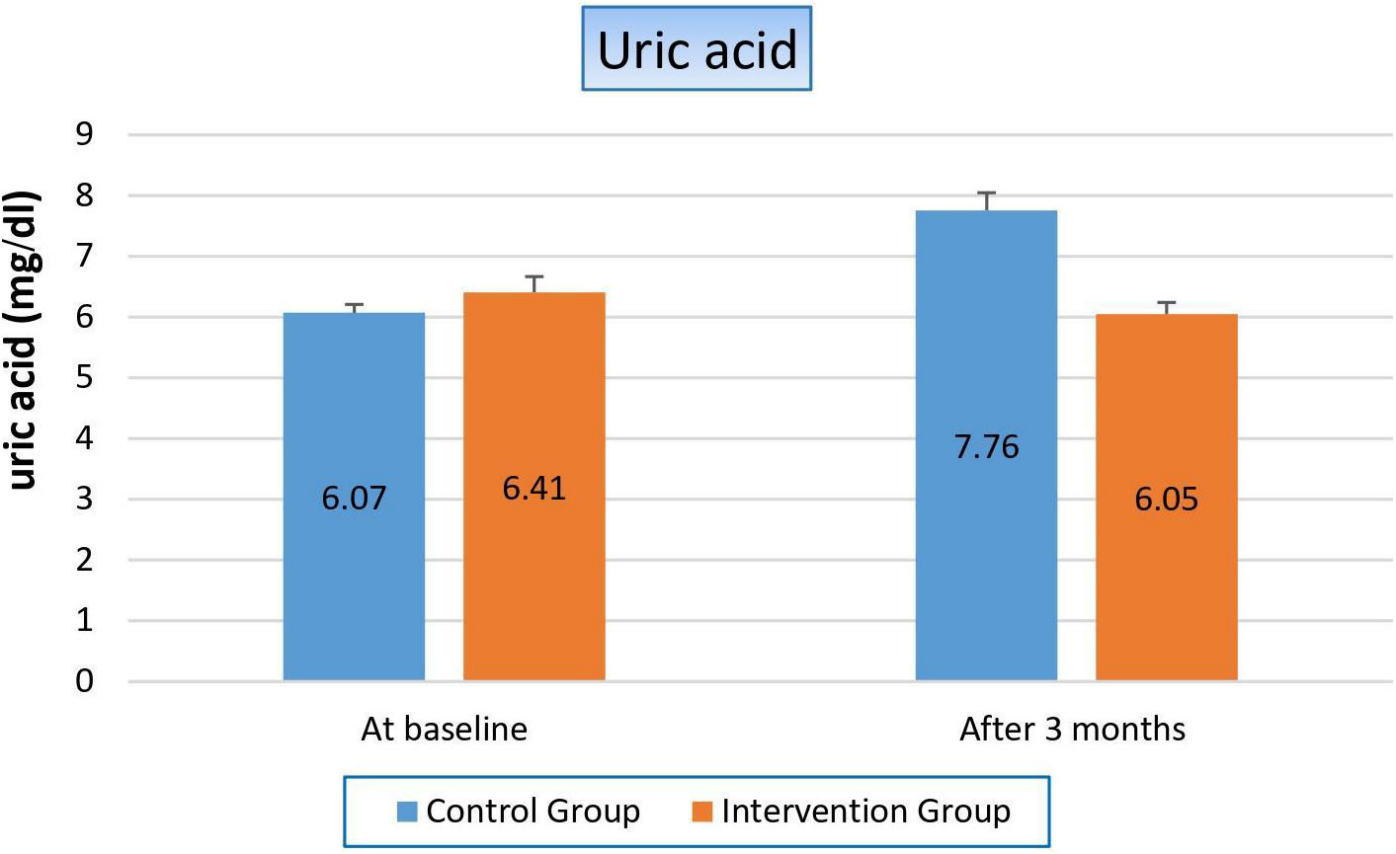
Changes in uric acid levels in control versus intervention groups at baseline and after 3 months.

There was severe deterioration in uric acid level in the control group, which increased by 27.8% after 3 months (*p*-value 0.000), while it slightly decreased in the niacin group but with a non-significant value (*p*-value 0.189). These results made the comparison between both groups decided in Favor of the niacin group, as shown in Figure 4 and Table 6.

**TABLE 6:** Effect on uric acid.

Variable	Study groups		
	Control group	Intervention group	*P*-value
**Uric acid (mg/dl)**			
At baseline Mean ± SE	6.07 ± 0.139	6.41 ± 0.257	0.250
After 3 months Mean ± SE	7.76 ± 0.288	6.05 ± 0.192	**0.001**
T-statistics	−4.373	1.353	
*P*-value	**0.000**	0.189	

Bold values indicate statistically significant results (*p* < 0.05).

#### 3.4.4 Effect on parathyroid hormone

**FIGURE 5:**
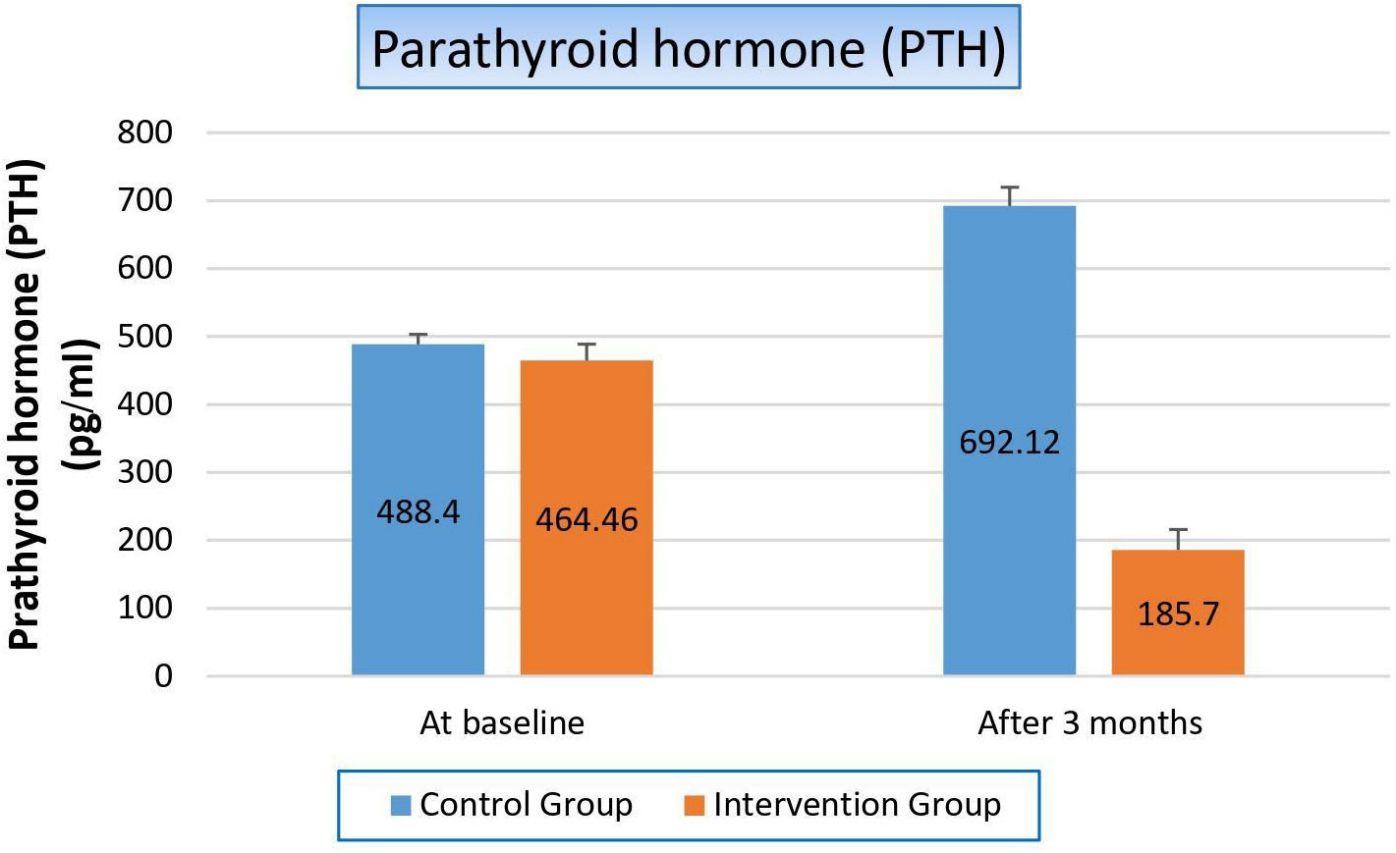
Changes in parathyroid hormone levels in control versus intervention groups at baseline and after 3 months.

Figure 5 and Table 7 showed that there was hyperparathyroidism in all study populations as a result of renal failure, but it was more worsened in the control group than in the niacin group, where it increased by 41.6% from the baseline result in the control group, while it decreased by 60% after 3 months from the start of niacin therapy in the treated group. Which reflected significantly on the *p*-value between both groups (*p*-value 0.001).

**TABLE 7:** Effect on parathyroid hormone.

Variable	Study groups		
	Control group	Intervention group	*P*-value
**Parathyroid hormone**			
At baseline Mean ± SE	488.40 ± 14.821	464.46 ± 24.282	0.059
After 3 months Mean ± SE	692.12 ± 27.614	185.70 ± 30.259	**0.001**
T-statistics	−6.746	−4.372	
*P*-value	**0.000**	**0.000**	

Bold values indicate statistically significant results (*p* < 0.05).

#### 3.4.5 Effect on lipid profile

**FIGURE 6:**
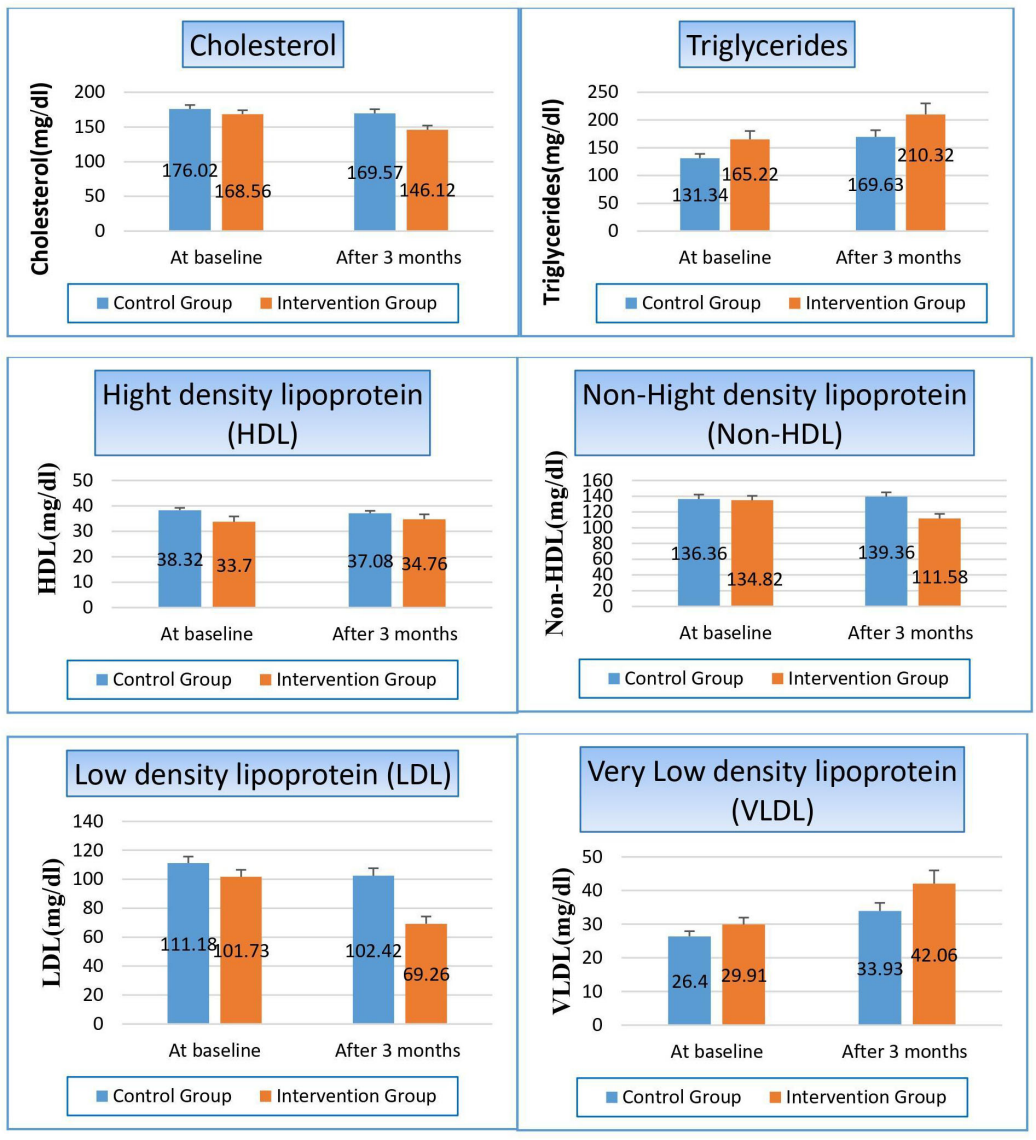
Changes in lipid profile in control versus intervention groups at baseline and after 3 months.

There were some parameters significantly changed, such as cholesterol, while the other parameters were not affected, such as triglycerides. Cholesterol was reduced in both groups, but with a higher percentage on the patients in the niacin group than on the patients in the control group (13.3% and 3.7%, respectively), with a *p*-value of 0.008. Non-HDL was significantly changed in both groups, where it increased by 2.2% in the control group while it decreased by 17.23% in patients treated with niacin, which significantly affected the p-value between both groups (*p*-value 0.001). Also, we observed the reduction of LDL levels in both groups, but with a higher percentage on the patients in the niacin group, where the percent reduction was 31.9% and 7.9%, respectively (*p*-value 0.000). Conversely, VLDL increased significantly in both groups but also with a higher percentage in the niacin group, where the percent increase was 40.6 and 28.5%, However, there was a difference of statistical significance between the two groups (*p*-value 0.138). The same results were observed in TG results, which increased significantly in both groups, especially in the niacin group, where they increased by 45.1 and 38.29 mg/dL, respectively, without affecting the *p*-value between both groups (*p*-value 0.135), as shown in Figure 6 and Table 8.

**TABLE 8:** Effect on lipid profile.

Variable	Study groups		
	Control group	Intervention group	*P*-value
**Cholesterols (mg/dl)**			
At baseline Mean ± SE	176.02 ± 5.683	168.56 ± 5.576	0.353
After 3 months Mean ± SE	169.57 ± 6.032	146.12 ± 5.981	**0.008**
T-statistics	5.103	3.879	
*P*-value	**0.000**	**0.000**	
**Triglycerides (mg/dl)**			
At baseline Mean ± SE	131.34 ± 7.760	165.22 ± 15.096	0.051
After 3 months Mean ± SE	169.63 ± 12.088	210.32 ± 19.701	0.135
T-statistics	−3.372	−2.112	
*P*-value	**0.000**	**0.035**	
**High density lipoprotein (HDL) (mg/dl)**			
At baseline Mean ± SE	38.32 ± 0.899	33.70 ± 2.139	0.055
After 3 months Mean ± SE	37.08 ± 0.978	34.76 ± 1.906	0.111
T-statistics	−2.162	−0.822	
*P*-value	**0.031**	0.411	
**Non-HDL (mg/dl)**			
At baseline Mean ± SE	136.36 ± 5.655	134.82 ± 5.649	0.848
After 3 months Mean ± SE	139.36 ± 5.513	111.58 ± 5.963	**0.001**
T-statistics	−13.25	3.728	
*P*-value	**0.000**	**0.001**	
**Low density lipoprotein (LDL) (mg/dl)**			
At baseline Mean ± SE	111.18 ± 4.508	101.73 ± 4.810	0.158
After 3 months Mean ± SE	102.42 ± 5.221	69.26 ± 5.016	**0.000**
T-statistics	5.233	6.952	
*P*-value	**0.000**	**0.000**	
**Very low-density lipoprotein (VLDL) (mg/dl)**			
At baseline Mean ± SE	26.40 ± 1.537	29.91 ± 2.062	0.053
After 3 months Mean ± SE	33.93 ± 2.417	42.06 ± 3.940	0.138
T-statistics	−3.029	−3.466	
*P*-value	**0.002**	**0.002**	

Bold values indicate statistically significant results (*p* < 0.05).

#### 3.4.6 Effect on lipoprotein a

**FIGURE 7:**
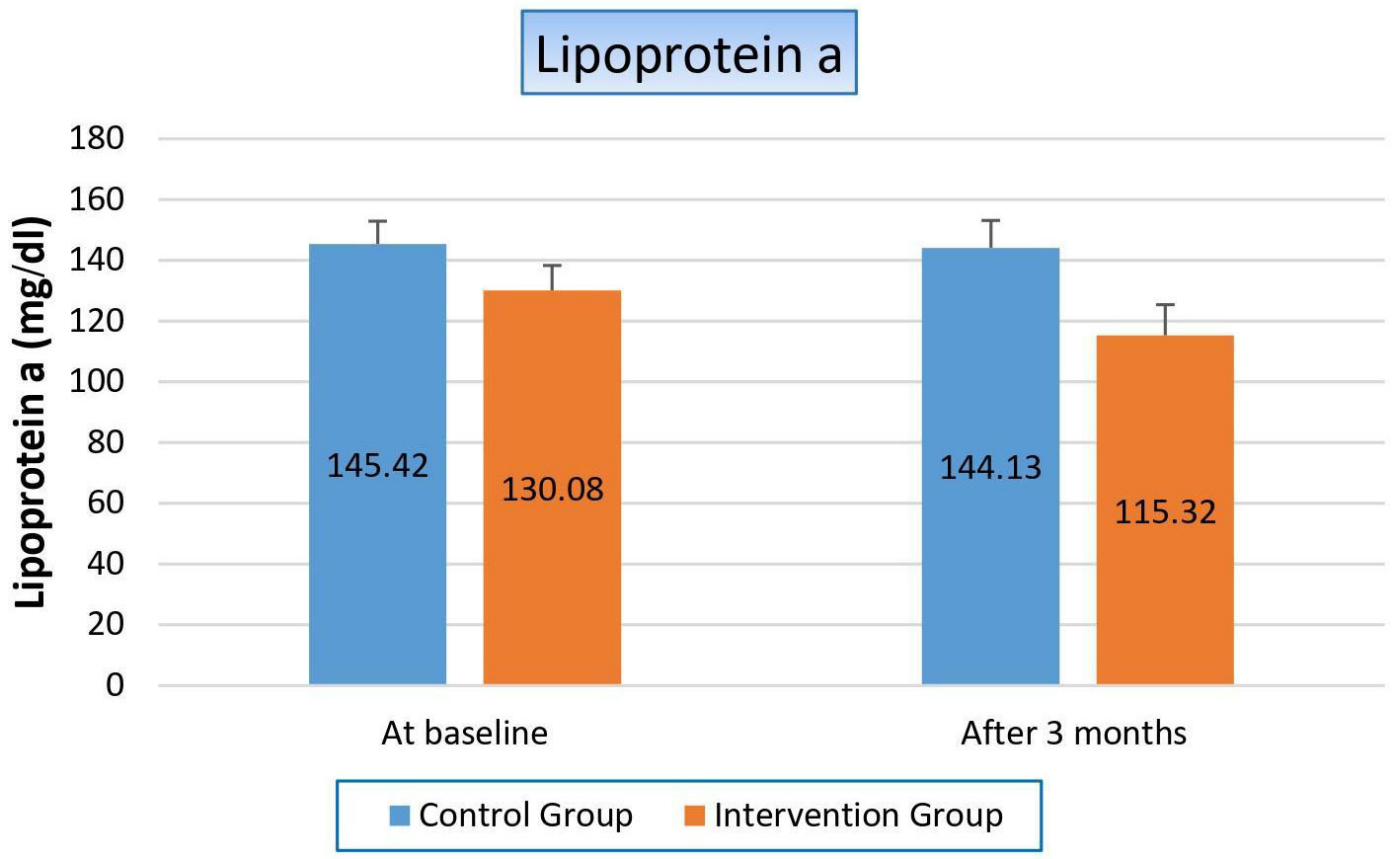
Changes in serum lipoprotein (a) levels in control versus intervention groups at baseline and after 3 months.

Finally, the way niacin affected blood levels of lipoprotein (a) staggered us, the key marker in our study, where it significantly decreased by 14.78 mg/dl (11.4%) in the treated group (*p*-value 0.003), while its value was not affected within the control group (*p*-value 0.721). So, the *p*-value significantly changed when we compared both groups with each other (*p*-value 0.038), as shown in Figure 7 and Table 9.

**TABLE 9:** Effect on lipoprotein a.

Variable	Study groups		
	Control group	Intervention group	*P*-value
**Lipoprotein a (mg/dl)**			
At baseline Mean ± SE	145.42 ± 7.473	130.08 ± 8.239	0.174
After 3 months Mean ± SE	144.13 ± 9.017	115.32 ± 10.089	**0.038**
T-statistics	0.361	3.257	
*P*-value	0.721	**0.003**	

Bold values indicate statistically significant results (*p* < 0.05).

## 4 Discussion

Mortality and morbidity associated with ESRD are primarily due to CVD, particularly on dialysis. As well, hyperphosphatemia is considered one of the most common complications of ESRD and is particularly predominant in people on HD. Elevated blood phosphorus levels have been associated to calcific uremic artery disease, metastatic calcifications, secondary hyperparathyroidism, and chronic kidney disease-mineral bone disorder (CKD-MBD). Patients with ESRD, including those undergoing HD, face an elevated risk of morbidity and mortality linked to hyperphosphatemia. This condition notably contributes to increased cardiovascular complications and related mortality in this population. Niacin has been shown to lower the level of phosphorus on the blood in patients with HD and CKD in previous studies. In our study, we compared lipoprotein a marker as one of the latest biomarkers in this field to the other investigational tests when assessing the dual effects of niacin on lipid profile and serum phosphorus in dialysis patients.

Our study was applied to 50 patients in the National Institute of Urology & Nephrology (NIUN), Cairo, Egypt, in dialysis units. Patients were randomly assigned into two equal groups of 25 each: the control group, which received standard therapy alone for 3 months, and the niacin group, which received 500 mg of niacin daily alongside standard therapy over the same period.

At a baseline, both groups were matched regarding their comorbid conditions, potential causes of hemodialysis, their clinical data, and laboratory tests. Which means the well-randomization process and complete homogeneity of both groups, so any further difference that appears at the end of the study will be due to our intervention.

In regard to blood pressure (BP), both systolic and diastolic blood pressure significantly improved in the niacin group as compared to the control group, could be due to several mechanisms through which niacin (vitamin B3) may influence cardiovascular health. The 1st mechanism is its vasodilation effect, which lowers blood pressure by increasing the capacity of blood vessels to carry blood, reducing the resistance against which the heart has to pump. The 2nd mechanism is attributed to niacin’s favorable impact on endothelial function, the inner lining of blood vessels. Enhanced endothelial activity improves vascular dilation and constriction capacity, thereby contributing to the regulation and maintenance of normal blood pressure. The 3rd one is its anti-inflammatory properties. Where chronic inflammation is a known contributor to cardiovascular disease and elevated blood pressure. By reducing inflammation, niacin could help reduce vascular resistance and lower blood pressure.

There were only two studies that determined the effect of niacin on BP: the study of Crystal A. Gadegbeku et al. and Kelly et al.

Our results are in line with those that were published by Crystal A. Gadegbeku et al., who used pulse waveform analysis to examine the hemodynamic effects of nicotinic acid (NA) infusion in 10 hypertensive and 11 normotensive patients. According to their research, hypertensive people’s mean blood pressure significantly decreased from 105 ± 2 mmHg to 100 ± 3 mmHg (*P* < 0.01), and their systolic, diastolic, and pulse pressures also significantly decreased (21).

In contrast, our findings differed from those of Kelly et al., who investigated the potential effects on blood pressure of nicotinic acid (NA) therapy-induced short-term impairment in insulin sensitivity. Seven healthy volunteers (four women and three men) participated in their double-blind, randomized, placebo-controlled crossover trial. They were given either a placebo or NA at a dose of 500 mg per day for 7 days, followed by 1 g per day for seven more days. The results showed that NA had no significant effect on 24-hour mean systolic or diastolic blood pressure. But this study suffered from a very small sample size with a short duration (22).

Also, we studied the effect of niacin on serum electrolytes. Not only on phosphorus level, as in previous studies, but also on potassium and sodium levels. Regarding phosphorus level, patients within the control group suffered from a significant hyperphosphatemia by 11.9% (*p*-value 0.008). On the other hand, there was a significant improvement in phosphorus level within the niacin group, where it reduced from 5.59 to 4.85 to become within normal range (*p*-value 0.0077). These findings may be attributed to the ability of niacin to inhibit intestinal phosphate absorption and lower parathyroid hormone (PTH) levels. Similar results were reported by Ahmed et al., who conducted a double-blind randomized clinical trial involving 50 hemodialysis patients over 6 months and demonstrated that niacin significantly reduced serum phosphate levels in this population. Consistent evidence has also been provided by other studies, including those of Edalat-Nejad et al. and Schepers et al., which confirmed that both niacin and niacinamide effectively lower phosphate concentrations in dialysis patients. Thus, niacin may be considered a safe and effective adjunct therapy in the management of hemodialysis patients. (10, 23, 24).

The same observation was noticed in potassium level, where controlled patients gained a significant hyperkalemia, which increased by 23.9% from baseline, while there was a significant reduction in potassium level in the niacin group by 27%, so there was a highly significant reduction between both groups in favor of the niacin group. The observed significant reduction in potassium levels in the niacin group, contrasted with increased hyperkalemia in controls, is less frequently reported but plausible given niacin’s impact on potassium metabolism, possibly through reduced intestinal absorption or enhanced excretion. This novel finding suggests niacin’s potential benefit for hyperkalemia management in hemodialysis patients, which is an important clinical consideration.

On the contrary, hyponatremia increased in the control group by 4.13%, while the decrease in sodium level was still within the normal range in the niacin group, which significantly appeared in the comparison between both groups (*p*-value 0.000). In the niacin group, the sodium levels remained within the normal range, indicating that niacin might have played a role in maintaining electrolyte balance. While niacin is not directly known for its effects on sodium regulation, its broader impact on improving overall metabolic and renal functions could have indirectly contributed to stabilizing sodium levels. The significant difference between the two groups (*p*-value 0.000) highlights the effectiveness of niacin in preventing the worsening of hyponatremia compared to the control group. However, the lack of significant changes within each group suggests that the variations were more pronounced when comparing the groups rather than within them individually. The literature on niacin’s direct impact on sodium levels in hemodialysis patients is limited, with most studies focusing on its lipid-modifying or phosphate-lowering effects. Some studies on niacin in CKD patients have reported changes in sodium, but these are often secondary findings or not consistently significant, as in Najafabadi et al. study, they carried out a double-blind randomized clinical trial in hemodialysis patients treated with niacin, where the dosage was gradually escalated from 50 mg to 100 mg per day in two monthly stages. Following the intervention, a significant increase in serum sodium levels was observed (*P* < 0.05). This novel finding in our study warrants further investigation to understand the underlying mechanisms and clinical implications of niacin’s role in sodium balance in ESRD (25).

Niacin’s effect on uric acid demonstrated that there was severe deterioration in uric acid serum level in the control group, which increased by 27.8% after 3 months (*p*-value 0.000), this exacerbation of hyperuricemia in the control group is consistent with the natural progression of renal dysfunction, where impaired uric acid excretion leads to its accumulation. Conversely, the niacin group showed a slight, though non-significant, decrease in uric acid levels (*p*-value 0.189). The highly significant difference between the two groups (*p*-value 0.000) underscores the stark contrast in outcomes, favoring the niacin group in terms of preventing a sharp rise in uric acid levels. However, the non-significant change within the niacin group suggests that its effect on uric acid might not be robust or consistent across all individuals. These findings add new evidence to the field, as no prior studies directly evaluated niacin’s effect on uric acid in this population, making our findings novel.

Concerning the effect of niacin on parathyroid hormone (PTH), there was hyperparathyroidism in all study populations as a result of renal failure. Hyperparathyroidism is a common consequence of renal failure due to the kidneys’ reduced ability to excrete phosphate and activate vitamin D. This leads to elevated parathyroid hormone (PTH) levels as the body attempts to compensate for imbalances in calcium and phosphorus metabolism. Therefore, there was more deterioration in the control group than in the niacin group. Where parathyroid hormone increased by 41.6% from the baseline result in the control group, while it decreased by 60% after 3 months from the start of niacin therapy in the treated group. Which reflected significantly on the *p*-value between both groups (*p*-value 0.001). The notable improvement in the niacin group resulted from its action on the reduction of serum phosphorus levels by inhibiting intestinal phosphate absorption, which in turn decreases the stimulus for PTH secretion. This explains the remarkable 60% reduction in PTH levels observed in the niacin group after 3 months of treatment.

Our results confirmed the results of two previous clinical trials Ahmed et al. and Najafabadi et al. studies who found that niacin caused a statistically significant decrease in level of PTH in dialysis patients (10, 25). However, our results were in contrast with the results of Edalat-Nejad et al. and Malhotra et al. studies who demonstrated no significant change in serum PTH levels (23, 26).

Niacin is well-known for its lipid-lowering properties, particularly its ability to reduce cholesterol levels. In our study, we studied its effect on each marker of lipid profile individually.

After 3 months, both the control group (*P* = 0.000) and the niacin-treated group (*P* = 0.000) showed a statistically significant reduction in total cholesterol levels. More importantly, the intergroup comparison after 3 months revealed a highly significant difference favoring the niacin group (*P* = 0.008), indicating a superior effect of niacin in lowering serum total cholesterol levels.

In the niacin group, the higher percentage reduction in cholesterol (13.3%) compared to the control group (3.7%) can be attributed to niacin’s mechanism of action. Niacin inhibits the synthesis of very-low-density lipoprotein (VLDL) and low-density lipoprotein (LDL) cholesterol in the liver, which leads to a significant decrease in circulating cholesterol levels. We observed this effect on the level of LDL, which reduced in both groups but with a higher percentage in the niacin group, where percent reduction was 31.9% for niacin group and 7.9% for control group (*p*-value 0.000). The significant reduction observed in the niacin group, coupled with the highly significant intergroup difference, underscores the robust effect of niacin on this critical lipid parameter. These findings were matched with Ahmed et al. and Ha Phan et al. studies (10, 27).

On the other hand, VLDL increased significantly in both groups but also with a higher percentage in the niacin group, where the percent increase was 40.6% for Niacin group and 28.5% for control group, but the intergroup comparison after 3 months did not reveal a statistically significant difference (*P* = 0.138). The significant increase in VLDL levels in both groups, with a higher percentage in the niacin group, could be attributed to niacin’s complex effects on lipid metabolism. While niacin is effective in reducing LDL cholesterol and triglycerides, it can also stimulate the production of VLDL particles in some cases. This paradoxical effect might explain the observed increase in VLDL levels in the niacin group. The exact mechanisms behind this increase are not fully understood and may vary depending on individual metabolic responses. We considered the 1st study showed its effect on VLDL, and there were no previous studies that showed these results.

Despite niacin’s established role in improving lipid profiles by lowering LDL cholesterol, triglyceride and raising HDL cholesterol, some patients may paradoxically experience elevated triglyceride (TG) levels after prolonged treatment. In our study, TG levels increased significantly in both groups, particularly in the control group, where levels rose by 29% for control group and 27.3% for niacin group. However, the intergroup comparison after 3 months did not reveal a statistically significant difference (*P* = 0.135). The aforementioned result was in line with Cheng et al. study, where TG levels increased in the niacin treated group but the increases was not significant (28). But this finding was in contrast with Ahmed et al. study, where TG levels decreased in the niacin treated group (10).

This paradoxical TG elevation may be attributed to mechanisms such as niacin-induced insulin resistance, which enhances free fatty acid flux to the liver (22), promoting hepatic triglyceride synthesis and VLDL secretion (29). Additionally, chronic inhibition of hepatic diacylglycerol acyltransferase-2 (DGAT2) may disrupt lipid homeostasis, triggering compensatory TG production (30). Furthermore, genetic variability and lifestyle factors, including diet and physical activity, may modulate these effects, underscoring the importance of individualized monitoring during niacin therapy.

Regarding HDL, our study observed the following changes: the control group showed a statistically significant reduction in HDL (*P* = 0.031), while the niacin-treated group showed an increase in HDL, but the change was not significant (*P* = 0.411). The intergroup comparison after 3 months did not reveal a statistically significant difference (*P* = 0.111). Niacin is well-known for its ability to increase HDL-C levels, primarily by reducing the catabolism of HDL apolipoprotein A-I (apoA-I) and inhibiting hepatic HDL uptake. This result was similar to that reported by Edema et al. study (31).

In the current study, the control group showed a statistically significant increase in Non-HDL (*P* = 0.000), while the niacin-treated intervention group exhibited a statistically significant reduction (*P* = 0.001). The intergroup comparison after 3 months revealed a highly significant difference favoring the niacin group (*P* = 0.001), indicating a superior effect of niacin in lowering non-HDL levels. Non-HDL cholesterol is considered a strong predictor of cardiovascular risk, as it includes all atherogenic lipoproteins. The significant reduction in non-HDL in the niacin group is a positive finding and aligns with niacin’s known ability to reduce atherogenic lipoproteins. The observed increase in non-HDL in the control group further emphasizes the progressive nature of dyslipidemia in hemodialysis patients without intervention. Our findings suggest that niacin can effectively improve this crucial cardiovascular risk marker in this patient population. We considered the 1st study showed its effect on non-HDL, and there were no previous studies that showed these results.

Lp(a) is a key cardiovascular risk marker, so its reduction is associated with a reduction in cardiovascular complications, which are considered the cornerstone of high morbidity and mortality in ESRD. Therefore, we focused on the effect of niacin on it. In our study, the treated group experienced a significant decrease in Lp(a) levels (11.4%, *p*-value 0.003), while the control group showed no significant change (*p*-value 0.721). When comparing the two groups, the *p*-value of 0.038 indicates a statistically significant difference between the effects of niacin and the lack of intervention in the control group. This difference underscores niacin’s efficacy in reducing Lp(a) levels, which is not commonly achieved by other lipid-lowering therapies. The significant reduction in Lp(a) levels in the niacin-treated group, compared to the control group, highlights niacin’s unique ability to target this specific lipoprotein by inhibiting its hepatic production. This finding suggests that niacin could play a unique role in cardiovascular risk management, particularly for patients with high baseline Lp(a) levels who are at an elevated risk for cardiovascular events, as in the case of our study population. This aligns with our study’s goals; the results could pave the way for larger clinical trials or further exploration of niacin as a therapeutic option. We considered the first study to determine the niacin effect on Lp(a) in HD patients, whereas previous studies primarily focused on diabetic or cardiovascular disease patients, or non-hemodialysis CKD patients, as in Sahebkar et al., Kalil et al. and Marshall B. Elam et al. studies (7, 32, 33).

Compared with conventional phosphate binders and lipid-lowering agents, niacin offers a distinctive therapeutic profile. Calcium-based and non-calcium binders effectively reduce serum phosphorus, but are often limited by hypercalcemia risk, gastrointestinal side effects, or high cost. Statins are widely prescribed to manage dyslipidemia in dialysis patients, yet they have minimal impact on Lp(a), which is increasingly recognized as an independent cardiovascular risk factor. In contrast, our findings show that niacin not only improved lipid parameters, including a significant reduction in Lp(a), but also lowered serum phosphorus and PTH, suggesting pleiotropic benefits that extend beyond conventional therapies. Given its relatively low cost, niacin may serve as a practical adjunct to standard binders and statins, particularly in patients with persistent hyperphosphatemia or elevated Lp(a). However, larger and longer-term studies are required to determine whether niacin could be positioned as a replacement or as a cost-effective alternative in selected hemodialysis populations.

In our study, no serious adverse events were reported; however, we did monitor for common niacin-related side effects such as flushing, gastrointestinal upset, hepatotoxicity, and hyperglycemia. The absence of clinically significant events may be attributed to the relatively low dose used (500 mg/day), the use of extended-release tablets and the short treatment duration (3 months). Nevertheless, we acknowledge that higher doses and long-term administration could increase the risk of hepatotoxicity, worsening glycemic control, and hyperuricemia, as previously reported in the literature. This addition strengthens the clinical relevance of our findings by balancing efficacy with safety considerations. Nevertheless, larger and longer-term studies are required to better characterize the safety profile of niacin in dialysis patients and to optimize dosing strategies that balance efficacy with tolerability.

### 4.1 Strengths and limitations

This randomized controlled trial has several strengths. It is among the few clinical studies to simultaneously evaluate the effects of niacin on lipid metabolism, Lp(a), phosphorus, uric acid, and PTH in hemodialysis patients. The randomized design minimized selection bias and ensured balanced baseline comorbidities between groups, thereby strengthening internal validity. All laboratory assessments were performed in a single central laboratory using standardized protocols, which reduced inter-assay variability and enhanced consistency across participants. Importantly, niacin demonstrated a favorable effect on Lp(a)—a lipid marker largely unresponsive to statins—while also improving markers of mineral metabolism. Furthermore, adverse events were systematically monitored, and no major toxicities were reported during the 3-month trial, supporting the short-term safety and feasibility of niacin in this population.

At the same time, several limitations should be acknowledged. First, the study’s somewhat modest sample size and single-center design limit how broadly the results may be applied. Second, the follow-up period was limited to 3 months; while sufficient to detect significant biochemical changes, this duration does not allow assessment of long-term sustainability or translation into clinical outcomes such as hospitalization, cardiovascular events, or mortality. Third, although standardized laboratory methods were used within our center, differences in assay methodology across institutions may limit external comparability. Fourth, while the reductions in phosphorus and PTH are attributed mainly to inhibition of intestinal phosphate absorption, additional mechanisms such as modulation of vitamin D metabolism and fibroblast growth factor-23 cannot be excluded. Fifth, although adverse events were systematically assessed, the relatively short trial duration may not capture the full spectrum of niacin-related toxicities, including hepatotoxicity, hyperglycemia, and gout flares, which may emerge with longer exposure. Sixth, the absence of disaggregated analyses by sex, age and comorbidities is a further limitation. Although baseline comparisons showed no significant differences in age, gender distribution or comorbidities between the niacin and control groups, the relatively small sample size prevented meaningful exploration of sex, age or comorbidities-specific responses. Finally, formal cost-effectiveness analysis was not performed, although niacin’s low cost compared with non-calcium phosphate binders and novel lipid-lowering agents makes it a potentially attractive option in resource-limited settings.

### 4.2 Recommendations

Although niacin exerted beneficial effects on multiple cardiovascular and metabolic risk markers, the extent of blood pressure improvement and triglyceride response may vary among individuals, requiring further mechanistic studies. Future research should incorporate larger sample sizes, extended follow-up durations, and multicenter designs to validate these findings and assess long-term sustainability, safety, and clinical outcomes. Subgroup analyses stratified by age, sex, and comorbidities are needed to clarify differential responses. Finally, formal pharmacoeconomic analyses will be essential to confirm whether niacin’s low cost translates into true cost-effectiveness in dialysis care.

## 5 Conclusion

Niacin (500 mg/day) demonstrated a significant dual effect in hemodialysis patients by improving both dyslipidemia and hyperphosphatemia. The treatment led to a statistically significant improvement in systolic and diastolic blood pressure, potentially attributable to niacin’s vasodilatory, endothelial, and anti-inflammatory mechanisms. Additionally, serum phosphorus levels were significantly reduced in the niacin group, contributing to a 60% decrease in PTH levels, thus mitigating secondary hyperparathyroidism.

The niacin group also exhibited a reduction in serum potassium and maintained sodium levels within the normal range, suggesting a stabilizing effect on electrolyte balance. While uric acid levels increased significantly in the control group, they remained stable with niacin treatment. Lipid profile improvements included significant reductions in total cholesterol and LDL-C. Although VLDL and triglycerides increased in both groups, intergroup differences were not statistically significant.

Importantly, niacin significantly reduced Lp(a) levels—an emerging cardiovascular risk marker—highlighting its potential in cardiovascular risk mitigation among ESRD patients. In light of these results, niacin may represent a low-cost and multifunctional adjunct that addresses both dyslipidemia and mineral metabolism disturbances. While these findings could ultimately inform future clinical practice guidelines, barriers such as flushing, hepatotoxicity, glucose metabolism disturbances, and the need for close biochemical monitoring must be considered. At present, niacin is best positioned as an adjunctive therapy, with its broader adoption contingent upon evidence from larger, multicenter, and longer-term trials.

## Data Availability

The original contributions presented in the study are included in the article/supplementary material, further inquiries can be directed to the corresponding authors.
